# Breast Cancer Cell Re-Dissemination from Lung Metastases—A Mechanism for Enhancing Metastatic Burden

**DOI:** 10.3390/jcm10112340

**Published:** 2021-05-27

**Authors:** Lucia Borriello, John Condeelis, David Entenberg, Maja H. Oktay

**Affiliations:** 1 Department of Anatomy and Structural Biology, Montefiore Medical Center, Einstein College of Medicine, Bronx, NY 10461, USA; lucia.borriello@einsteinmed.org (L.B.); john.condeelis@einsteinmed.org (J.C.); 2 Gruss-Lipper Biophotonics Center, Montefiore Medical Center, Einstein College of Medicine, Bronx, NY 10461, USA; 3 Department of Surgery, Montefiore Medical Center, Einstein College of Medicine, Bronx, NY 10461, USA; 4 Integrated Imaging Program, Montefiore Medical Center, Einstein College of Medicine, Bronx, NY 10461, USA; 5 Department of Pathology, Montefiore Medical Center, Einstein College of Medicine, Bronx, NY 10461, USA

**Keywords:** intravasation, dissemination, metastasis, TMEM doorways

## Abstract

Although metastatic disease is the primary cause of mortality in cancer patients, the mechanisms leading to overwhelming metastatic burden are still incompletely understood. Metastases are the endpoint of a series of multi-step events involving cancer cell intravasation, dissemination to distant organs, and outgrowth to metastatic colonies. Here we show, for the first-time, that breast cancer cells do not solely disseminate to distant organs from primary tumors and metastatic nodules in the lymph nodes, but also do so from lung metastases. Thus, our findings indicate that metastatic dissemination could continue even after the removal of the primary tumor. Provided that the re-disseminated cancer cells initiate growth upon arrival to distant sites, cancer cell re-dissemination from metastatic foci could be one of the crucial mechanisms leading to overt metastases and patient demise. Therefore, the development of new therapeutic strategies to block cancer cell re-dissemination would be crucial to improving survival of patients with metastatic disease.

## 1. Introduction

The mortality of breast cancer patients with localized disease has decreased considerably over the past 30 years as a result of earlier diagnosis and major advances in treatment [1]. In contrast, the outcome of patients with metastatic disease has not significantly improved, and approximately 90% of breast cancer-related deaths are due to distant metastases [2,3]. Metastasis is the endpoint of a complex sequence of events—collectively termed the metastatic cascade—whereby cancer cells in primary tumors intravasate into the blood vasculature and disseminate through the circulatory system to distant organs. These disseminated cancer cells (DCCs) then extravasate into the parenchyma of distant tissues where many survive in a dormant state before reinitiating proliferation to generate macroscopic, clinically detectable metastatic foci [4,5]. In contrast to the large body of findings that have revealed the detailed mechanisms leading to primary tumor formation, the biological underpinnings of metastatic disease remain poorly understood.

Although the traditional notion is that metastasis is a late event in tumor progression, increasing clinical and experimental evidence suggests that cancer cells can disseminate even before the primary tumor is clinically detectable [6]. These early DCCs colonize distant organs and survive there in a non-proliferative, dormant state [6]. These dormant DCCs are clinically undetectable, chemo-resistant, and immune privileged, which allows them to survive for years or even decades. Due to yet-to-be identified changes in the tissue microenvironment, DCCs eventually exit dormancy to resume proliferation, resulting in the formation of metastatic foci [7,8]. The latency and awakening of DCCs potentially explains the formation of metastasis years, or even decades, after resection of the primary tumor.

In addition to the well-established concept of tumor dormancy, a new mechanism that might contribute to overwhelming metastatic burden is the re-dissemination of cancer cells from metastatic foci to distant organs. The traditional view of metastatic progression [9] requires that cancer cells lose cell–cell junctions within the primary tumor, travel through the circulation, and colonize distant organs. However, the recent emerging concept of cancer self-seeding [10,11] has challenged this implicit view that metastasis is a uni-directional process. For instance, it has been demonstrated that cancer cells can disseminate in multiple directions, seeding not only secondary sites, but also re-seeding the primary site [10,11], as well as hematogenously disseminating from lymph nodes to seed tertiary sites [12,13,14]. The new metastatic model that has emerged is termed “metastasis from metastases” [10,11,15,16,17,18,19].

Using high-resolution multiphoton intravital imaging, our laboratory discovered that intravasation and dissemination of cancer cells from primary breast tumors occurs through specialized doorways named TMEM (Tumor MicroEnvironment of Metastasis) [20,21,22,23,24]. TMEM doorways are composed of three cell types that are in direct contact with each other [20,21,22,23,24]: a cancer cell that expresses high levels of Mena (Mena^high^) (an actin-regulatory protein that influences cell motility), a perivascular macrophage that expresses high levels of Tie2 and VEGF (Tie2^high^/VEGF^high^), and an endothelial cell. We have recently demonstrated that the opening of TMEM doorways depends on the release of VEGF from the Tie2^high^ perivascular macrophage, which in turn causes a local disruption of the underlying endothelial junction proteins, Zonula Occludens-1 and Vascular-Endothelial Cadherin [20,25]. Migrating cancer cells intravasate through the resulting transient opening of the endothelial wall and enter the blood circulation to disseminate to secondary sites [20,25]. TMEM doorways form primarily on blood vessels and not on lymphatic vessels, in both primary tumors and metastatic foci in lymph nodes [12]. Importantly, TMEM doorways have been identified in primary breast tumors resected from patients [21,22,23,26] and the density of TMEM doorways can be used as a prognostic marker for distant recurrence in breast cancer patients [21,22,26]. Recently, we have demonstrated the presence of TMEM doorways in human lung and lymph node metastases from breast cancer patients [12,27]. We have named these S-TMEM (secondary-tumor microenvironment of metastasis) doorways. The presence of functional S-TMEM doorways in lung and lymph node metastases suggest that re-dissemination of cancer cells might occur from secondary sites via the same TMEM doorway mediated mechanism that is present in primary tumors. This would allow cancer cells to make their way back to the primary tumor site, as well as to re-disseminate to tertiary sites. Though never conclusively proven, this concept is supported by clinical evidence that in some patients, circulating cancer cells are found in the blood years or even decades after the removal of the primary tumors [28,29] The presence of circulating cancer cells is a prognostic for tumor recurrence and poor survival [30,31], suggesting that these cancer cells would be causative for further metastasis in tertiary sites and for hastening the death of cancer patients.

Here, using photo-convertible fluorescent proteins to fate-map cells that originate from metastases, we conclusively show, for the first time, that breast cancer cells are able to re-intravasate and re-disseminate from lung metastases to seed tertiary sites. Our findings provide a new potential explanation for enhanced metastatic progression in certain patients, and support the concept that cancer cell intravasation remains a clinically relevant therapeutic target, even after resection of the primary tumor. Thus, understanding the molecular mechanisms of cancer cell re-dissemination will provide therapeutic targets to improve survival of patients with metastatic disease.

## 2. Materials and Methods

### 2.1. Cell Culture

E0771 medullary breast adenocarcinoma cells, originally isolated from a spontaneous mammary tumor in C57BL/6 mice, were obtained from Dr. Wakefield’s lab at the NIH, who in turn obtained them from Dr. Fengzhi Li in Dr. Enrico Mihich’s lab at Roswell Park Cancer Institute, Buffalo, NY. The E0771 stably expressing-Dendra2 were generated with standard lentiviral transfection procedures and were FACS sorted for the over-expression of Dendra2. Cancer cells were cultured in DMEM (cat #12320032, ThermoFisher, Grand Island, New York, USA) media supplemented with 10% (*v/v*) FBS (cat# S11550, R&D System, Flowery Branch, Ga, USA) and 1% penicillin/streptomycin (cat# 15140122, Gibco, Grand Island, NY, USA) and were routinely tested for mycoplasma (LookOut Mycoplasma PCR detection kit, cat #MO0035-1KT, Sigma, St. Louis, MO, USA). 

### 2.2. Animals

All procedures were conducted in accordance with the National Institutes of Health regulation concerning the care and use of experimental animals and with the approval of the Einstein College of Medicine Animal Care and Use Committee (IACUC). The immunocompetent wild type C57BL/6 female mice between 12 and 24 weeks of age were used for the experiments. The mice were originally purchased from Jackson laboratories.

### 2.3. Experimental Metastasis

E0771-Dendra2 cells were prepared by trypsinizing a 10 cm confluent culture dish of cancer cells and passing them through a 40 μm cell strainer (Falcon, cat #352340, Corning, Rochester, NY, USA) to avoid clumps. A total of 1 × 10^6^ cells were re-suspended in 50 μL of sterile PBS and intravenously injected via the lateral tail vein. Three weeks post-injection mice developed lung metastases. The presence of lung metastases in all experimental mice was confirmed by H&E staining of harvested lung tissue at the end of the experiment (Supplemental Figure S1). 

### 2.4. Photoconversion

Mice bearing lung metastasis, prepared as described above using E0771-Dendra2 cells, were anesthetized and intubated. To surgically open the chest cavity, a 1 cm incision was made on the right side of the chest through the skin and the intercostal muscle in the chest wall, as previously described [32]. Extreme care was taken to ensure that surgical manipulation was made only to the skin and intercostal muscles, and that the lung tissue, and the metastases they harbor, were not touched during surgery, minimizing the risk of iatrogenic spread of cells. An aluminum foil mask was affixed to the anesthetized animal, shielding the body from light-exposure while limiting illumination, and thus accomplishing photoconversion solely to the lung tissue of interest, as previously published [12]. Photoconversion of lung metastases was accomplished by 4 min of continuous illumination using a custom built 400 nm light emitting diode array lamp with the lamp (cat# 897-LZPD0UA00, LedEngin Inc., San Jose, CA, USA) [33,34] set to its highest intensity. The chest wall was then closed with 5–0 sutures and the mouse extubated. Mice with lung metastases that did not undergo photoconversion were used as controls. Three days after photoconversion, mice were sacrificed, and blood, brain, liver, and bone marrow were collected to check for the presence of red-photoconverted cancer cells.

### 2.5. Processing of Samples and Detection of Photoconverted Cancer Cells

For blood collection, mice were anesthetized with isoflurane and about 1 mL of blood was drawn from the right heart ventricle using 25G needles coated with heparin. Erythrocytes were lysed using 10 mL of 1× RBC lysis buffer (cat# 00-4333-57, eBioscience, Carlsbad, CA, USA) for 10 min at room temperature. Blood samples were centrifuged at 1000 rpm for 5 min and cells were reconstituted in 5 mL of DMEM supplemented with 10% FBS, plated in a 35 mm glass-bottom dish, and allowed to adhere overnight.

Brain was harvested and washed with PBS. Then, the organ was cut into small pieces and digested with 10,000 collagenase digestion units/mL of collagenase I (cat# C0130, Sigma, St. Lous, MO, USA), 32 mg/mL of Dispase II (cat# 4942078001, Millipore, Mannheim, Germany), and 5 MU/mL of DNase I (cat# 260913, Millipore, Billerica, MA, USA) in PBS for 2 h at 37 °C, as previously described [35]. The resulting single-cell suspension was washed with PBS supplemented with 0.5% (*w*/*v*) BSA and filtered through a 70 mm nylon mesh. Cells were resuspended in 5 mL of DMEM supplemented with 10% FBS, plated in a 35 mm glass-bottom dish, and allowed to adhere overnight.

Bone marrow was flushed from both left and right femurs using 25G needles, and erythrocytes were lysed using 1 mL of 1× RBC lysis buffer for 10 min a room temperature. Bone marrow samples were centrifuged at 1000 rpm for 5 min, cells were reconstituted in 5 mL of DMEM supplemented with 10% FBS, plated in a 35 mm glass-bottom dish, and allowed to adhere overnight.

The following day, cells were washed with PBS three times, permeabilized with 0.1% (*v/v*) Triton X-100 for 5 min, and stained with DAPI (cat# SKU FP1490, Akoya Biosciences, Marlborough, MA, USA) for 5 min. Fluorescence images were captured using an epi-fluorescence microscope (DeltaVision Core, General Electric, Boston, MA, USA) with a 60× objective, and CoolSNAP HQ2 CCD camera (Photometrics, Tucson, AZ, USA). The standard excitation/emission filter sets for FITC and TRITC were used to visualize green and red fluorescence, respectively. The threshold for determining photoconversion (red signal) was based on the autofluorescence of cells in non-tumor bearing mouse. Any cells showing red signal above that threshold were considered positive. These cells were confirmed to be tumor cells by the simultaneous presence of green fluorescence. We did not observe any cells with red signal in the organs taken from non-photoconverted mice.

## 3. Results

**Figure 1:**
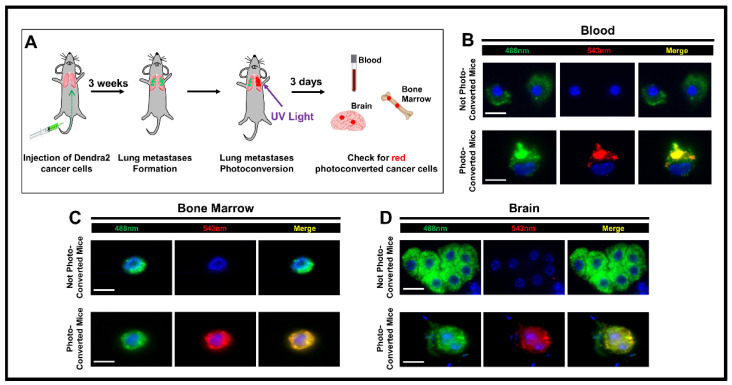
Cancer cells disseminate from lung metastases to tertiary sites. (**A**) Experimental design. E0771 Dendra2-expressing cancer cells are injected into the tail veins of C57BL mice to generate lung metastases. Approximately three weeks later, lung metastases are exposed to UV light for photoconversion. Three days after photoconversion, blood, brain, and bone marrow were collected and analyzed for the presence of red-fluorescent cancer cells. (**B**–**D**) Representative images of cancer cells detected in the blood, brain, and bone marrow of mice. Top panels are images of disseminated cancer cells (DCCs) with non-photoconverted Dendra2 (note green-fluorescence) from control mice (no photoconversion), whereas the bottom panels show images of DCCs from mice with photoconverted lungs (note green- and red-fluorescence). Scale bar 10 µm.

Based on our previous data showing the presence of functional S-TMEM doorways in lung metastases [27], we postulated that cancer cells might re-intravasate and re-disseminate from metastatic foci in the lung to tertiary sites. To test this hypothesis, we employed a method that allowed us to determine the site of DCC origin. This strategy utilizes the photoconvertible fluorescent protein, Dendra2, as described previously [12,36]. Dendra2 is a green light-emitting protein that can emit in the red spectrum upon exposure to 400 nm light [12,36,37]. E0771 breast cancer cells stably expressing Dendra2, were intravenously injected into syngeneic C57BL/6 mice to generate lung metastases, as previously described [27]. Three weeks post-injection, we opened the chest and exposed the lung metastases to light from a custom built 400 nm light-emitting diode array lamp to convert Dendra2-positve cancer cells from green to red fluorescence, while limiting ultraviolet light exposure of the lungs. Three days post-photoconversion, we determined whether red cells could be detected in tertiary sites, indicating intravasation and dissemination from the lungs (Figure 1A. The presence of red-photoconverted cells in these organs proves that these cells originate from lung metastases and disseminated after the photoconversion. In addition to cells that generated only green fluorescence (which could have either re-disseminated from the lung metastases prior to photoconversion or were cells which lodged in these organs upon initial tail vein injection), we indeed found red-photoconverted DCCs in the blood, brain, and bone marrow of mice that had undergone photoconversion, clearly indicating that these DCCs originated from lung metastases (and not from other sites) and were able to seed tertiary sites (Figure 1B–D). To rule out the possibility that cancer cells undergo spontaneous photoconversion, we collected blood, brain, liver, and bone marrow from mice that had not undergone photoconversion. As expected, we found only green DCCs (Figure 1B–D bottom panels), indicating that cancer cells do not go through spontaneous photoconversion, and that any red cancer cells originated specifically from lung metastases. To eliminate the possibility that some host cells may produce a confounding signal similar to photoconverted DCCs, we photoconverted lungs of mice without established metastases in their lungs and looked for the presence of green and red fluorescent cells in the extracted organs. We did not observe cells expressing either green or red fluoresce signal in the blood, brain, or bone marrow. However, we did observe cells expressing both greed and red fluoresce in the liver. Upon antibody staining we identified these cells as macrophages. Given the difficulty of distinguishing whether fluorescent cells in the liver are tumor cells or macrophages, we excluded the liver from our analyses. Indeed, it was previously documented that some cell types, such as macrophages, may show autofluorescence [38].

Our data thus indicate that breast cancer cells are capable of re-intravasating in established lung metastases and re-disseminating to other distant organs.

## 4. Discussion and Conclusions

Despite the progress in cancer treatments, metastasis remains the main cause of cancer patient mortality. Metastases are the endpoint of a dynamic and complex series of events during which cancer cells intravasate and disseminate to distant sites where they eventually form secondary tumors. The majority of studies investigating the development of metastases have focused on the steps that occur after extravasation, such as awakening of dormant DCCs and outgrowth of the secondary tumors. Other potential mechanisms that might explain the formation of overwhelming metastatic burden have not been investigated due to the lack of appropriate technologies.

In this short communication, we used a novel technology that allowed us to selectively photoconvert metastatic cells in the lung and track their fate. Here, for the first time, we show that breast cancer cells are able to enter the blood vasculature from lung metastases, re-disseminate, and seed tertiary organs. Our data suggest that breast cancer cell dissemination occurs not only from the primary tumor site but also from metastatic sites, which may perpetuate metastatic dissemination even after the removal of the primary tumor, potentially enhancing metastatic burden and causing death of cancer patients. Metastases may therefore arise from awakening of disseminated dormant DCCs that might have disseminated from primary tumor and/or re-dissemination of DCC from metastatic foci.

**Figure 2:**
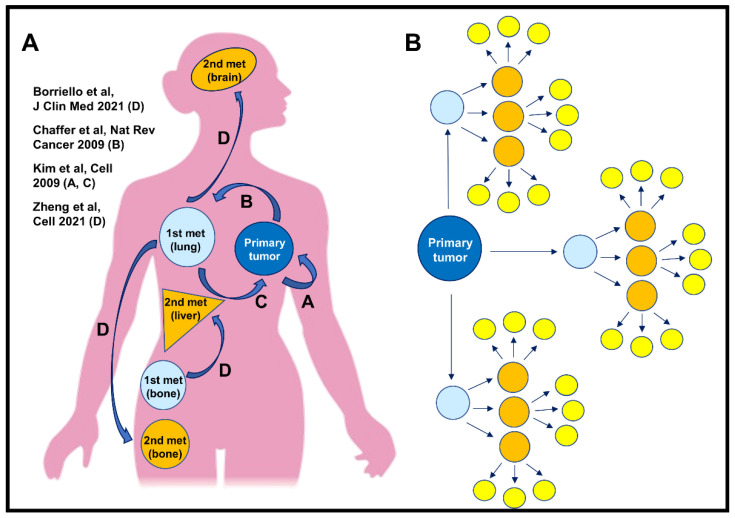
The roads of metastasis. (**A**) Cartoon representing multi-directional pathways of metastasis formation. Pathway A. Cancer cells can leave the primary tumor, circulate, and eventually return to its origin, i.e., primary tumor [10]. Pathway B. Cancer cells can leave the primary tumor and extravasate at the secondary site (e.g., lungs) where they form metastatic foci [4,5]. Pathway C. From a metastatic focus, cancer cells can also re-intravasate and re-seed back to the primary tumor (if still in place), or travel and seed tertiary metastatic sites, Pathway D [12,13,14,39], enhancing metastatic burden. (**B**) Schematic representation showing that cancer cell re-dissemination from metastatic foci drastically enhances metastatic burden, which may contribute to cancer patient mortality. Primary tumor that, according to the classic concept of metastasis, produce only three metastatic foci (light blue circles), could, after only two rounds or re-dissemination originating from these three metastatic foci, result in 27 new metastatic foci (yellow circles), provided that the dissemination rate is constant. Thus, cancer cell re-dissemination from metastatic foci may be a new mechanism of enhancing metastatic burden.

Our data are in accordance with the new concept that metastasis is a multi-directional process [11]. In this process, multiple pathways could lead to metastases (Figure 2). For example, cancer cells could disseminate and then return to the primary tumor bed (pathway A). Another cancer cell might follow a similar pathway, but instead colonizes a metastatic site and forms a metastatic nodule (pathway B). Cells within this nodule, such as from the bone marrow metastases [39] could additionally re-intravasate and re-seed back to the primary tumor, if still in place (pathway C). Finally, cells within this nodule could re-disseminate to tertiary metastatic sites (pathway D) (Figure 2A). 

Previously, Kim et al., using a variety of cancer cell lines (breast, colon, and melanoma), demonstrated how disseminated cancer cells can colonize their tumors of origins (pathway A and C) [10]. Recently, we and others (using a similar technological advance), have shown that cancer cells can disseminate from lymph nodes to seed tertiary sites such as the lung [12,13]. Here, for the first time, we demonstrate that a new dissemination path exists: one in which cancer cells within established lung metastases re-intravasate and seed tertiary sites (pathway D). If cancer cells which re-disseminate from metastatic foci are capable of initiating growth at tertiary sites, re-dissemination would induce an exponential growth in the number of nodules (Figure 2B). For example, if the primary tumor produces only three metastatic foci and each focus subsequently produces additional three metastatic foci, after only two rounds of re-dissemination there would be 27 metastatic foci (Figure 2B, yellow circles).

The precise mechanism of cancer cell re-dissemination from metastases, including studies to determine if the re-dissemination occurs via S-TMEM doorways is warranted. The observation of functional TMEM doorways in lung metastases [27] demonstrates that they are not restricted to the primary tumor microenvironment but also implicates them in cancer cell dissemination from metastatic sites. As such, TMEM doorways may be a common mechanism of cancer cell dissemination, present at all tumor sites and during all stages of breast cancer progression. Therefore, the clinical observation that circulating cancer cells can be found in the blood of patients years or decades after removal of their primary tumor [28,29] may mean that S-TMEM doorways perpetuate dissemination throughout the disease course and multi-directionally. Thus, it would not be too late to inhibit TMEM function, even after the removal of the primary tumor, because in some patients, cancer cells might have already disseminated from the primary site and formed clinically undetectable micro-metastases, which are a source of further metastatic cancer cell dissemination. That is, inhibiting TMEM doorway function at metastatic sites even after the removal of the primary tumor could block dissemination of cancer cells from metastatic sites, thereby decreasing overall metastatic burden and extending patient survival. Moreover, given that most patients with metastatic disease receive some form of chemotherapy, and that chemotherapy can increase the density and activity of TMEM doorways in some patients [40,41], combining chemotherapy with TMEM doorway function inhibitors may improve outcomes in patients with metastatic disease. Several such trials are already underway (NCT02824575, NCT03717415).

In conclusion, our study demonstrates that metastasis from metastases occurs, and supports the notion that pharmacological inhibition of cancer cell dissemination may be necessary, even after resection of the primary tumor, to control metastasis. We conclude that it is “never too late” to block dissemination when treating cancer patients.

## Data Availability

The raw data supporting the conclusions of this article will be made available by the authors.

## Supplementary Materials

The following are available online at https://www.mdpi.com/article/10.3390/jcm10112340/s1, Supplemental Figure S1. Immunohistochemistry of E0771-Dendra2 lung metastasis. Representative low- and high-power images of hematoxylin and eosin-stained lung metastasis (**A**,**D**) 1 week, (**B**,**E**) 2 weeks, and (**C**,**F**) 3 weeks post-injection.
