# Supplementary material for: Breast Cancer Cell Re-Dissemination from Lung Metastases—A Mechanism for Enhancing Metastatic Burden
Source: J Clin Med. 2021 May 27;10(11):2340. doi: 10.3390/jcm10112340 (PMC8199463; doi:10.3390/jcm10112340)

Figure S1. Immunohistochemistry of E0771-Dendra2 lung metastasis. Representative low- and high-power images of hematoxylin and eosin-stained lung metastasis (**A**,**D**) 1 week, (**B**,**E**) 2 weeks, and (**C**,**F**) 3 weeks post-injection.

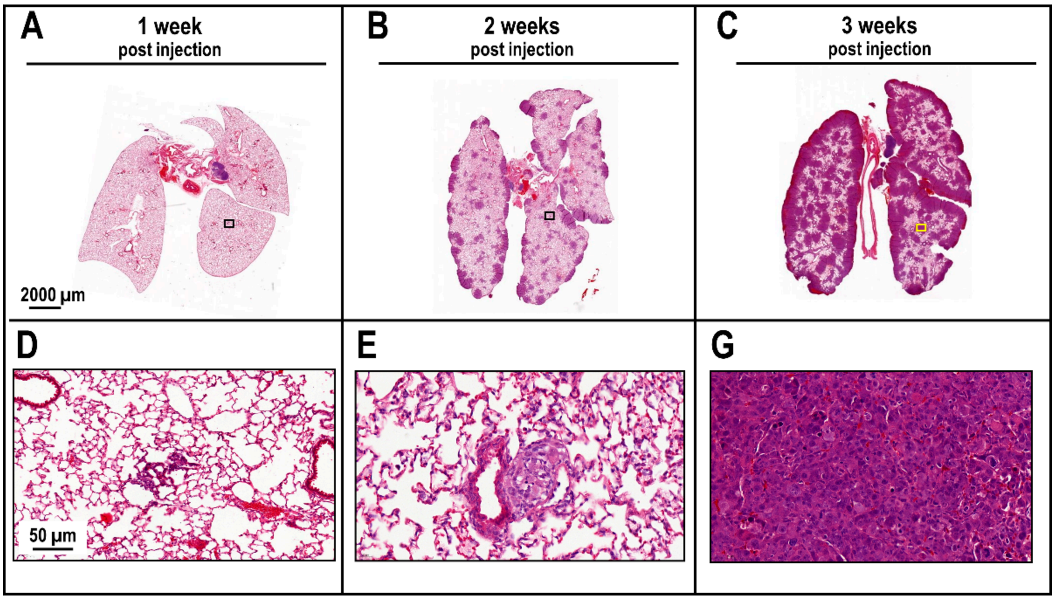

Supplement: Supplementary file 1 [file jcm-10-02340-s001.zip › jcm-1215496-SI.pdf]
